# Relaxation Response in Stressed Volunteers: Psychometric Tests and Neurotrophin Changes in Biological Fluids

**DOI:** 10.3389/fpsyt.2021.655453

**Published:** 2021-06-17

**Authors:** Miriam Zappella, Filippo Biamonte, Bijorn Omar Balzamino, Rocco Manieri, Magdalena Cortes, Daniela Santucci, Enrico Di Stasio, Maurizio Rizzuto, Alessandra Micera

**Affiliations:** ^1^Department of Psychology, Salesian University of Rome, Rome, Italy; ^2^Department of Neuroscience, IRCCS Children's Hospital Bambino Gesù, Rome, Italy; ^3^Department of Basic Biotechnological Sciences, Intensive and Perioperative Clinics, Catholic University of the Sacred Heart, Rome, Italy; ^4^Research and Development Laboratory for Biochemical, Molecular and Cellular Applications in Ophthalmological Sciences; IRCCS – Fondazione Bietti, Rome, Italy; ^5^Department of Laboratory and Infectivological Sciences, UOC Chemistry, Biochemistry and Molecular Biology Clinic, Fondazione Policlinico Universitario A. Gemelli IRCCS, Rome, Italy; ^6^Hebrew Hospital Rome Ophthalmology Department, Rome, Italy; ^7^Prevention and Health Care Department, Campus Bio Medico University, Rome, Italy; ^8^Cellular Biology and Neurosciences, Istituto Superiore di Sanita, Rome, Italy

**Keywords:** PSS-10 perceived stress scale, nerve growth factor, brain derived neurotrophic factor, tears analysis, saliva analysis, relaxation response meditation technique

## Abstract

**Background:** To evaluate the beneficial effects of relaxation response (RR) training in adult stressed subjects by evaluating the psychometric response recorded at relaxation session. Cortisol as well as nerve growth factor (NGF) and brain derived neurotrophic factor (BDNF) mediators were quantified in both saliva and tears, and their levels were related to each other and to the psychometric response.

**Methods:** Stressed subjects (*n* = 23; 10M/13F; age range 21–53 years old) were voluntarily enrolled in the study. RR training sessions were carried out for 2 months, 1 day per week, at the same time (3–5 p.m.). Two different psychological questionnaires, the Perceived Stress Scale-10 (PSS-10) and the Beck Depression Inventory - Short Form (BDI-SF) and Ocular Surface Disease Index (OSDI) tests, were administered before each session. Saliva and tears were sampled for cortisol (EIA), NGF (ELISA), and BDNF (ELISA) quantifications. Questionnaires' data were analyzed and compared to biochemical ones.

**Results:** All subjects reported beneficial effects from training. RR significantly reduced the psychological stress indexes (*p* = 0.039 for PSS-10 and *p* = 0.001 for BDI-SF). Specifically, RR training lowered the perception of Perceived Helplessness (items 1, 3, 10; *p* < 0.05) in PSS-10 and increased the Perceived Self-Efficacy (*p* < 0.05). OSDI score was in the normal range (0–25). Biochemically, a decrease in cortisol, a trend to a decrease in NGF, and an increase in BDNF levels were observed in saliva samples after RR treatment. Furthermore, a trend to a decrease in NGF and an increase in BDNF were quantified in tear samples. A correlation between PSS-10 total score and saliva NGF variation (%) as well as between BDI-SF total score and BDNF tear levels were also observed.

**Conclusion:** RR training appeared useful to lowering psychological, mental, and physical stress, as supported by both psychological total and single scores. The finding on biochemical levels of BDNF in saliva and tears are sustained by previous studies while those of NGF require further investigation. Overall, these data on a small population highlight the potential use of RR training and potential neurotrophic changes in biological fluids, in stressed volunteers.

## Introduction

Stress is a pervasive phenomenon that increasingly concerns our society, representing a crucial aspect in both working and recreational activities, at any age (1). Many people experience at least once one-time high levels of stress. Persistent stress might trigger depression and anxiety, general pain, sleep and digestive problems, skin and eye conditions, metabolic impairments, and other mental/emotional health problems (2). A recent epidemiological study highlighted that a high percentage of deaths derives from stressful lifestyles (3).

The word “stress” refers to a variety of vegetative-emotional, cognitive, and behavioural phenomena occurring simultaneously when an organism is subjected to any adaptive task (4). Stress response is functional to maintain the homeostasis within organs and the tissue microenvironment, and resilient subjects respond better than anxious ones to stressors (5). Failures at both physiological and biochemical levels can occur as a result of uncontrolled hypothalamic-pituitary-adrenal (HPA) axis activation and adrenocorticotropic hormone (ACTH) release (6). Physiologically, circadian-mediated cortisol release exerts a negative feedback on ACTH, an effect not observed in the presence of chronic (pathological) release (7). Under stressful conditions, the huge release of circulating cortisol can play a critical role on immune-endocrine and visual systems, and in parallel, cortisol changes can over-tune the levels of stress by influencing the release of other neuromodulators (8).

In 1994, Aloe et al. reported for the first time the involvement of the prototype of the neurotrophin family, the nerve growth factor (NGF), in stress-driven responses: circulating NGF levels increased in young parachute soldiers experiencing their first jump (9). In the same way, another neurotrophin of the same family, the brain derived neurotrophic factor (BDNF), was observed in stress response, being modulated in response to acute stress with a compromised response to challenging situations (10–12). More recently, NGF and BDNF, either alone or in concert with cortisol, have been implicated in preventing care of mood and neurodegenerative disorders (13–15). Recently, a potential association between aggravating anxiety/depression and ocular surface discomfort was prospected (16). More precisely, severe symptoms of dryness were associated with high levels of anxiety in patients suffering from dry eye (17). In this context, both NGF and BDNF expressions were related to the pathology of dry eye disease (18).

In anxious subjects, relaxation response (RR) represents a non-invasive way to decrease the levels of circulating stressors (cortisol) and to increase resilience (19). Little data are available regarding this training and its possible modulation of circulating and tissue specific mediators (20).

Therefore, the main aim of the present study was to evaluate the beneficial effects of RR training in adult stressed subjects by analyzing the psychometric response recorded during training session. In addition, cortisol as well as NGF and BDNF mediators were quantified in both saliva and tears, and their levels were related to each other and to the psychometric response.

## Methods

### Reagents and Plasticwares

Sterile single package Schirmer's test strips were obtained from Accutome (Accutome, Inc., Phoenixville Pike, PA, USA) and sterile Salivette® devices came from Sarsted^TM^ (Milan, Italy). Plasticwares and analytical grade chemicals were purchased from EuroClone (Milan, Italy), Sigma Aldrich (St. Louis, MA, USA), ICN (Milan, Italy), and Carlo Erba (Milan, Italy), or elsewhere specified. RNAse-free demineralised MilliQ water was produced daily (Direct Q5 apparatus; Millipore Copr., Billerica, MA) and autoclaved according to standard procedure.

### Study Population

The study complied with ethical standards in the recruitment/treatment of participants and in the management of biochemical data. Study protocol was approved by the intramural review board of the Salesian University of Rome (Rome, Italy). Participants were informed about the purpose of the study and written informed consent was asked before test administration and saliva/tear sampling.

Twenty-three volunteers were enrolled to the study: 13 female (22–53 years old) and 10 male (22–45 years old) adults. Participants declared no systemic diseases, except for two females reporting, respectively hyperthyroidism and hyperglycaemia. The volunteers have been selected within a master classroom and the clinical evaluation was carried out by a senior psychologist (professor M.R.). All subjects completed the psychometric and Ocular Surface Disease Index (OSDI) questionnaires.

### Relaxation Response Sessions: Training Schedules, Psychological Questionnaire Structure, and Training Description

Training sessions (60 min per session) were carried out once a week for 2 months (between 15:00 and 17:00) to guarantee “circadian” reproducibility (21). The after-lunch choice was sustained by the low plasmatic cortisol level, allowing identification of any hypercortisolemia (22). RR training comprised two phases: the body relaxation phase and the memory/emotional phase, respectively. In the first phase, a study subject shall devote 12–15 min to this relaxation phase. It is necessary to choose a place without invasive disorders and to take a passive attitude toward the experience that will be made. Both calm and tranquility must be maintained until a study subject realizes that he or she is breathing abdominally. In the second phase, it is necessary for a study subject to keep his or her eyes closed, and to make mind to go to the memory of an episode where there was still no malaise and where the pain had not taken over. A study subject has to make that memory, playing with light, colors, sounds, focusing on as many details as possible. At this point, it is essential to turn subject attention to the future where that problem will no longer be there, such that the memory becomes as real as possible. Finally, the recovery exercise will be carried out (23).

Two different psychological questionnaires, the Perceived Stress Scale-10 (PSS-10) and the Beck Depression Inventory - Short Form (BDI-SF), were administered at the first and last sessions. For this study, PSS-10 included 10 items instead of the original 14 items (PSS-14), as 4 items (items 4, 5, 12, and 13) were removed due to a low factor relativity. PSS-10 questions are concerned with moods and thoughts related to the previous month. Subjects were asked how often they recognized themselves in such situation. The score range included: 1–10, below the average of perceived stress; 11–14, at average of perceived stress; 15–18, above average of perceived stress; and >19, an extremely high perceived stress score (24). BDI-SF, which consists of 13 items, has an internal consistency (rho 0.89–0.97) that is comparable to that of the long form, as well as a good sensitivity (97%), and an adequate specificity (77%), and has a cutoff score of 5 (25). BDI-SF was designed to assess the depth of depression, such that the score range included: 0–3, no depression; 4–7, light depression; 8–15, moderate depression; and >16, high depression (26). Both questionnaires were provided in validated Italian versions (24, 27). In addition, the OSDI questionnaire was administered shortly after the psychosocial ones, according to standard procedure (28).

### Saliva and Tears: Devices, Sample Preparation, and Assays

Saliva and tear samples were collected before and after each session by following an easy self-sampling procedure.

Saliva samples were collected by using Salivette® devices (Sarsted^TM^, Milan, Italy) and cotton-embedded samples, the devices were kept at room temperature and quickly centrifuged (13,000 rpm for 15 min; megafuge, Heraeus, Milan, Italy) to collect saliva, according to manufacturers' procedures. Finally, samples were stored at −70°C until analysis.

Tears were sampled by self-sampling after a brief demonstration by ophthalmologist (M.C.), or ophthalmologist's assistant if required. Schirmer's strips were placed only in the right eye for 2 min, as no patients suffering from dry eye were recruited (see OSDI data). Samples were quickly refrigerated and delivered to the laboratory (in a temperature-controlled box). Tear proteins were extracted from membranes, through shaking (300 rpm for 30 min; benchtop orbital shaker, Sigma), according to standard procedure including the extraction diluent, i.e., 10 mM phosphate buffer saline (PBS) with a neutral pH 7 supplemented with a cocktail of detergents and protease inhibitors. Cell debris were removed from extracts by centrifugation (13,000 rpm for 7 min; minifuge, Sigma Aldrich, Missouri, USA). Tear proteins were extracted from membranes, through shaking (300 rpm/30 min; benchtop orbital shaker, Sigma Aldrich, Missouri, USA), according to standard procedure including the extraction diluent PBS (10 mM phosphate buffer saline; pH 7) supplemented with a cocktail of detergents and protease inhibitors. Cell debris were removed from extracts by centrifugation (13,000 rpm for 7 min; minifuge, Sigma Aldrich, Missouri, USA).

### Biochemical Analysis: Cortisol, NGF, and BDNF

Both saliva and tear samples were spectrophotometrically analyzed (A280 program) to quantify total protein amounts in clarified samples (i.e., corpuscular component separated from liquid ones). (Nanodrop ND1000, Thermo Fisher Scientific Inc., Waltham, MA, USA). Extracted samples were used for enzyme immunoassay (EIA) or enzyme-linked immunosorbent assay (ELISA) depending on the candidate.

Cortisol quantification was carried out by using a human-specific chemiluminescent-based electrochemiluminescence immunoassay (ECLIA) (Roche Diagnostics S.p.A., Monza, Italy). Briefly, samples (10 μL each) were incubated with a specific biotinylated anti-cortisol antibody and a cortisol derivative labeled with a ruthenium complex. The reaction mixture was aspirated into the measuring cell where the microparticles were magnetically attracted to the electrode surface. Subsequently, the unbound substances were eliminated (ProCell/ProCell M). Results were calculated on a calibration curve generated specifically with a 2-point calibration and a master curve supplied with the reagent barcode or electronic barcode. All solutions were from the kit. Roche COBAS system was used for chemiluminescent signal acquisition. Streptavidin coated microparticles (at 0.72 mg/mL) reagents were used.

NGF and BDNF quantifications were performed by using double-sandwich ELISA assays. In short, DuoSet ELISA kits (R&D Systems, McKinley Place, Minneapolis, MN, USA) were employed for human βNGF (DY256-05) and human BDNF (DY248-05) detection in protein extracts from swabs and strips devices. Briefly, 96-well Maxisorp plates (Nunc, Roskilde, Denmark) were pre-coated with the specific capture antibodies (0.4 μg/mL; R&D) overnight (4°C). Samples were diluted 1:2 in assay diluent (from the kit; R&D) supplemented with 1x protease inhibitor cocktail (Pierce - Thermo Fisher Scientific Inc. Waltham, MA USA) and loaded in parallel with the standard curve (0.32–2,000 pg/mL protein; R&D). After an overnight incubation, the addition of the specific detection secondary antibodies (0.15 μg/mL; R&D) and streptavidin (1:200; R&D) were carried out. Specific binding was developed by using the ready-to-use TMB substrate and Stop solution (R&D). The colorimetric signals (Optic Density, OD) were acquired at λ 450–570 nm by using the Sunrise plate reader (Tecan Group Ltd., Männedorf, Switzerland). The related target values (pg/total protein amount; A280 Nanodrop analysis) were produced using a 3rd grade polynomial standard. Concentrations of analytes and data are shown as pg/mg (e.g., total protein), as calculated by GraphPad Prism 8.1 software (GraphPad Software Inc., San Diego, CA, USA). The absence of cross reactivity with other neurotrophins was declared by the manufacturer for both assays.

### Statistical Analysis

Comparisons were performed by using the Statistical Package for Social Sciences (SPSS) software Windows (Version 21, IBM, New York, NY, USA). Continuous variables were expressed as mean [±Standard Error of the Mean (SEM)] and categorical variables were displayed as frequency counts and percentages, respectively. A Kolmogorov-Smirnov normality test was performed to examine the distribution (29). Non-parametric tests were used to assess significant variations between subgroups. In particular, the Wilcoxon test was used to assess the effect of time (difference between paired data), while the Mann-Whitney *U*-test was conducted to evaluate differences between sexes (independent groups). With respect to the latter, for any subjects the sum of and the difference between the two repeated measures were computed, and the Mann-Whitney *U*-test was performed on these two derived variables. Significant differences between sexes on the sum or on the variation of repeated measures indicated significant main effect of sex or significant interaction sex^*^time, respectively. Correlations were calculated with Spearman correlation coefficient. A two-sided *p*-value ≤ 0.05 was considered for significance. Moreover, in addition to assessment of statistical significance, the effect size (“*d*” metric) was calculated for principal findings to measure the strength and clinical relevance of the relationship.

## Results

Subjects were exposed to RR training and collections of questionnaire data as well as fluid samples were performed at the first and last sessions, respectively.

### RR Training Modulates Both PSS-10 and BDI-SF Total Scores and Particularly Few PSS-10 and BDI-SF Items

Almost all study subjects declared a beneficial effect from RR sessions [first session: 22/23 (95.7%), and second session: 23/23 (100%)], despite individual variabilities. In the first session, a subject (1/23; 4.3%) declared no relaxation upon training (subject n.9, male, 45 years old) but stated benefits in the second one.

**Figure 1:**
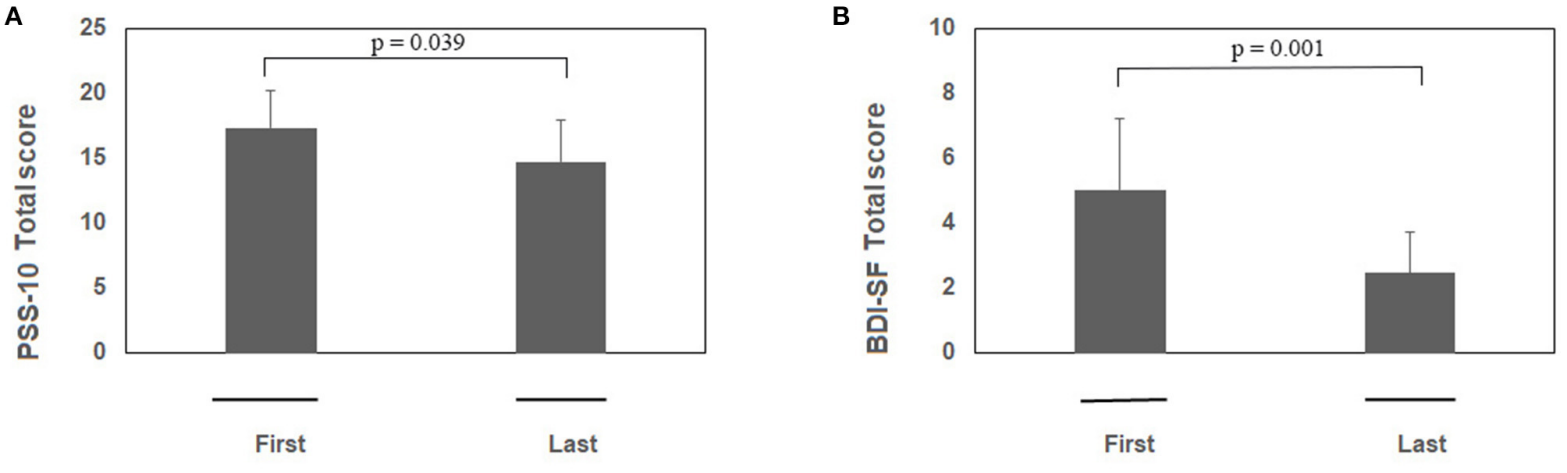
**(A,B)** RR training modulates both PSS-10 and BDI-SF total scores. Subjects were exposed to RR training and at the beginning of each session both questionnaires were administered. Histograms showing the PSS-10 **(A)** and BDI-SF **(B)** scores between first and last session during RR training (*n* = 23). Note the significant decreases in both PSS-10 and BDI-SF total scores between the pre first and pre last sessions during RR training. Data are presented as mean values (Mean ± SEM) and comparisons between groups were carried out by Wilcoxon test. Significance was set at *p* ≤ 0.05.

**Figure 2:**
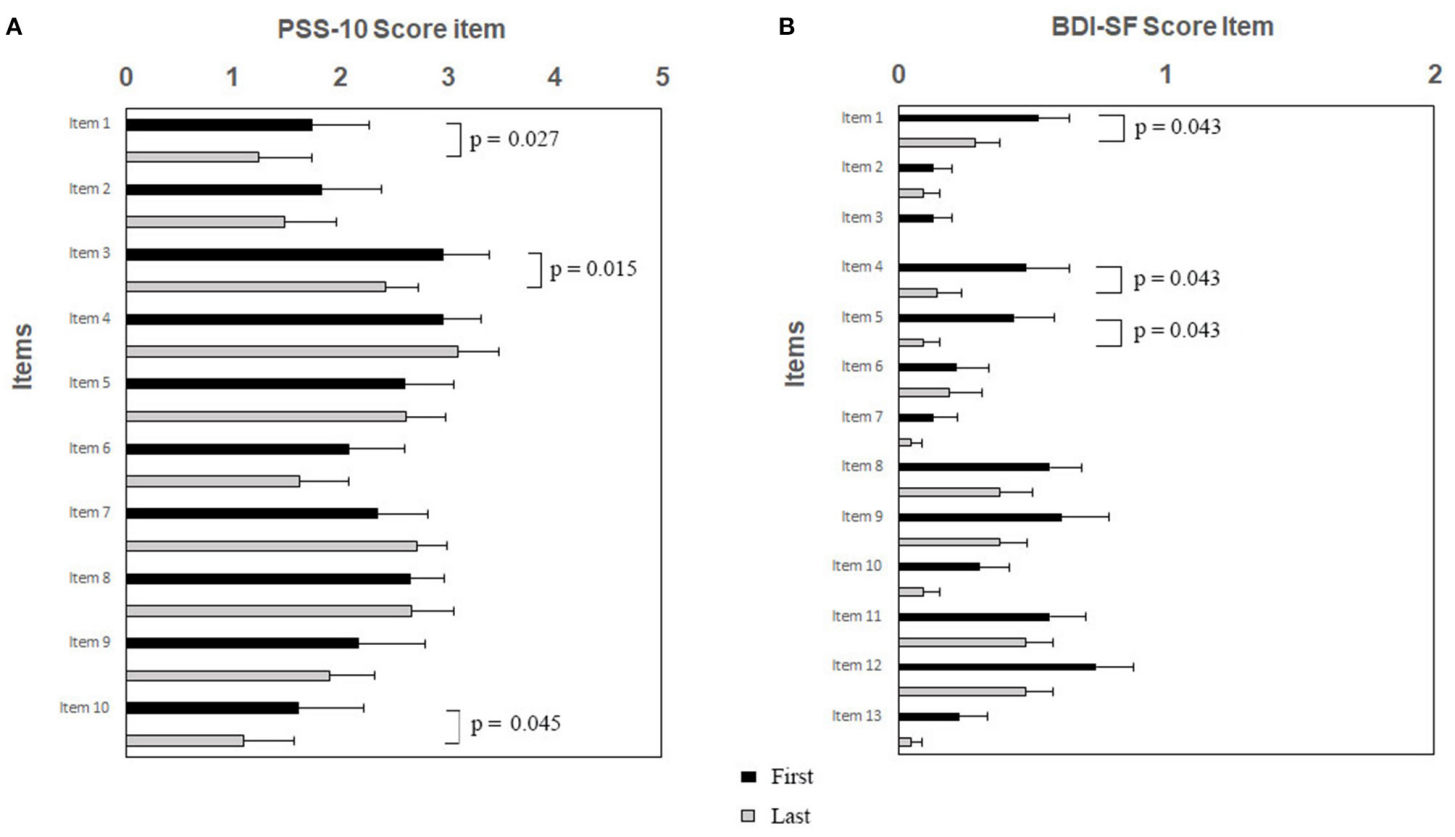
**(A,B)**. RR training modulates specific items of PSS-10 **(A)** and BDI-SF **(B)**. Subjects were exposed to RR training and at the beginning of each session both questionnaires were administered. Histograms showing specific PSS-10 and BDI-SF items are presented as black/gray bars indicate first/last sessions, respectively. Note the significant decreases of scores between sessions after RR training specifically for PSS-10 items 1, 3, and 10, and for BDI-SF items 1, 4, and 5, respectively. Data are presented as mean values (Mean ± SEM) and comparisons between groups were made by Wilcoxon test with a significance of *p* ≤ 0.05.

Psychological tests sustained the efficacy of RR training. The psychometric test total score is shown in Figure 1A (PSS-10) and Figure 1B (BDI-SF). The statistical analysis showed a decrease of PSS-10 total score between first and last sessions (17.3 ± 5.8 vs. 14.7 ± 6.5; *z* = −2.06; *p* = 0.039; effect size *d* = 0.40, small; Figure 1A). The same trend was observed for BDI-SF total score (5.0 ± 4.4 vs. 2.5 ± 2.5; *z* = −3.19; *p* = 0.001; effect size *d* = 0.67, medium; Figure 1B). Of interest, three out of PSS-10 (merely 1, 3, and 10) and three out of 13 BDI-SF (1, 4, and 5) items were significantly decreased (respectively, PSS-10: *z* = −2.20; *p* = 0.027; *z* = −2.43; *p* = 0.015; *z* = −2.00; *p* = 0.045; Figure 2A; and BDI-SF: *z* = −2.023; *p* = 0.043; *z* = −2.023; *p* = 0.043; *z* = −2.023, *p* = 0.043, Figure 2B). Moreover, a trend toward a decrease was observed for PSS-10 item 6 while an increase was noted for item 7, such that the constructs of these items were found to be inversely proportional. No gender dependent difference in the PSS-10 total score variation between first and last sessions was observed. The OSDI score distribution of observed patients was close to reference population range (0–25).

### Cortisol, NGF, and BDNF Levels in Saliva and Tears Were Modulated by RR Training

**Figure 3:**
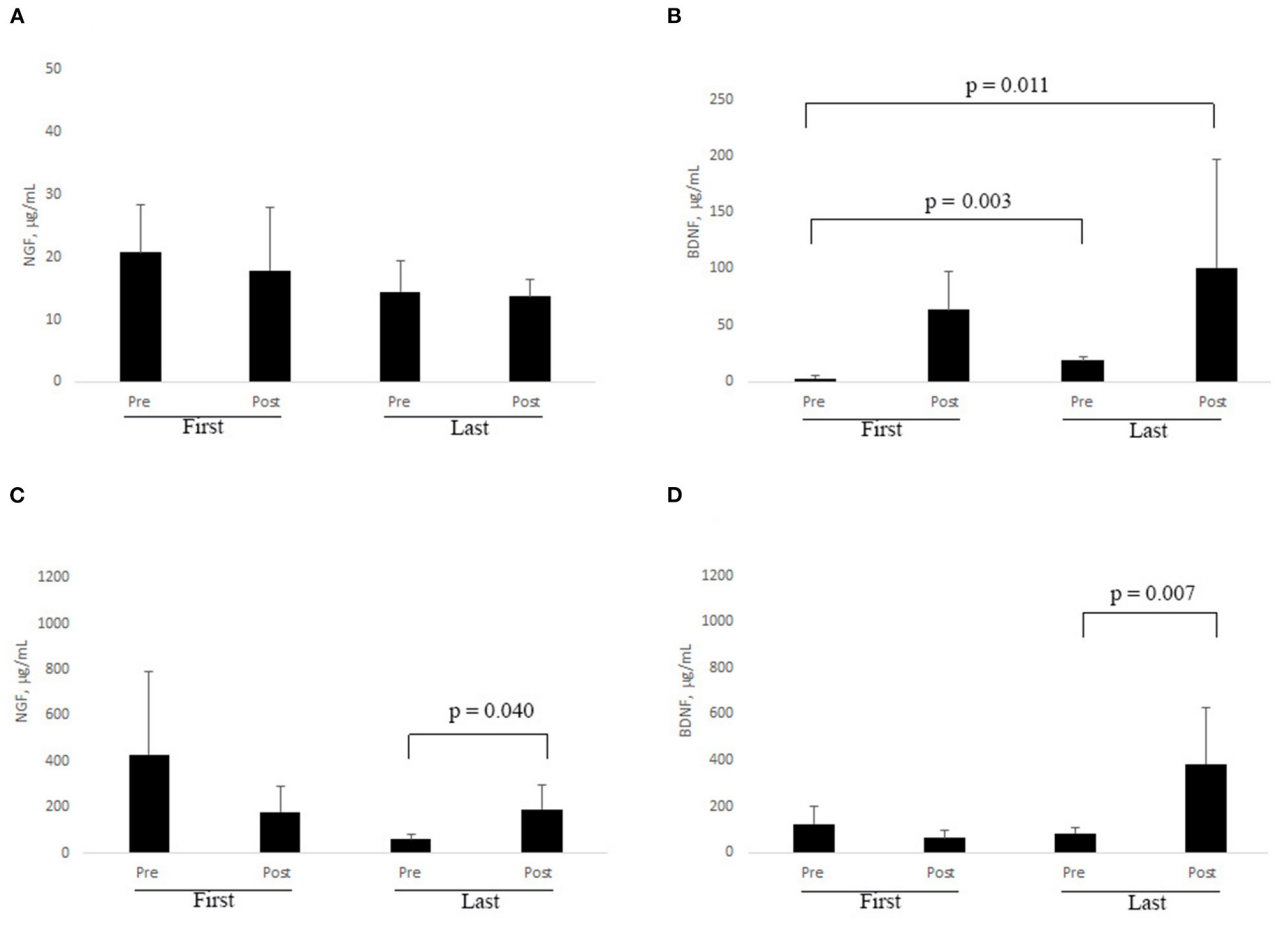
**(A–D)**. RR training influences NGF and BDNF levels in biological fluids. At each session of RR training, subjects were asked to provide saliva and tear samples by self-sampling (see Methods). ELISA results are shown for NGF in saliva **(A)** and tears **(C)**, and for BDNF in saliva **(B)** and tears **(D)**, respectively. Significant changes are indicated by *p* ≤ 0.05 (Non-parametric analysis: Wilcoxon test).

Eleven out of 23 subjects showed a trend to a decrease in cortisol level in saliva (0.115 ± 0.07 μg/dL vs. 0.094 ± 0.08 μg/dL). To ensure that the study subjects were at a comparable level of stress, the effects of environmental variation were minimized, such that within the study group, the cortisol levels of study subjects remained in the physiological range 0.054–0.138 μg/dL. Slight trends to a decrease of NGF and an increase of BDNF were observed in saliva samples after RR treatment. No evident changes were observed for NGF (Figure 3A) and BDNF (Figure 3B) in saliva and tears at first session (based on pre-post analysis). A comparison between pre first and post last sessions as well as between pre first and pre last sessions highlighted significant increases of BDNF in saliva samples (Figure 3B; *z* = −2.934; *p* = 0.003 and *z* = −2.521; *p* = 0.011, respectively).

Even if not statistically significant, a decrease of tear NGF was observed between pre and post last sessions, while an increase in the level of this neurotrophin was observed after the last one (*z* = −2.045; *p* = 0.040, Figure 3C). Analogously, a clear increase in BDNF level was monitored after the last RR session (*z* = −2.69; *p* = 0.007; Figure 3D).

**Figure 4:**
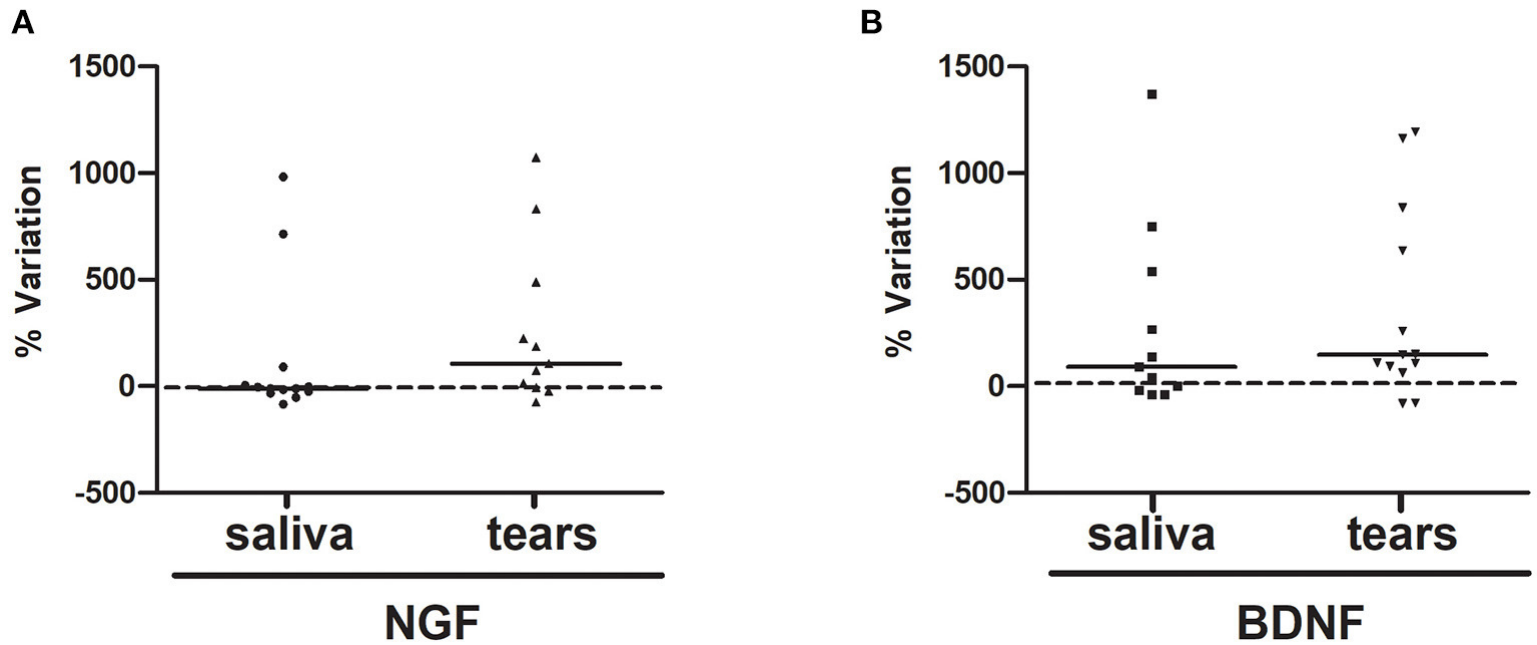
**(A,B)**. NGF and BDNF %variations upon RR training in the last session. The variations between pre and post values percentualized on pre value (post-pre)/pre*100 of NGF (**A**, saliva and tears) and BDNF (**B**, saliva and tears) were displayed by scatter plot.

The extents of concentration variability in the saliva and tear samples were assessed for both NGF and BDNF, as displayed by scatter plots (Figures 4A,B, respectively). In particular, due to possible large variability in the pre values, for any subject the variation between post and pre values has been standardized by perceptualizing it on the pre value [(post-pre)/pre^*^100].

A slight correlation between BDI-SF total score and BDNF tear level %variation was observed in the first session (rho = −0.97; *p* = 0.06; data not shown) while, in the last session, PSS-10 total score fully correlated with saliva NGF %variation (rho = 0.59; *p* = 0.05). Moreover, when considering post session NGF and BDNF values, PSS-10 total score slightly correlated with both neurotrophin levels in saliva in the last session (rho = −0.50; *p* = 0.06; rho = −0.57; *p* = 0.06, respectively) while BDI-SF total score was clearly correlated with NGF saliva levels in both first and last post sessions (rho = 0.66; *p* = 0.009; rho = 0.31; *p* = 0.04). An additional correlation between first session BDI-SF total score and post first session BDNF tear level (rho = −0.97; *p* = 0.02) was also observed. Finally, a clear positive correlation was found between %NGF and %BDNF variation in both saliva (rho = 0.618; *p* = 0.05) and tear (rho = 0.845; *p* = 0.007) levels only in the last session. Finally, notable differences between sexes emerged in the BDNF levels when considering either the sum or the difference between pre and post salivary levels in the last RR session, with males showing higher protein levels than females (sum: *U* = 4.00, *z* = −2.00, *p* = 0.04 and difference: *U* = 5.00, *z* = −1.86, *p* = 0.06).

## Discussion

Herein, we provide evidence that RR training was effective in reducing the psychological test scores and the elevated levels of cortisol in saliva samples. In addition, RR training induced changes in NGF and BDNF levels in both saliva and tears.

Previous studies show that relaxation exercises and techniques, music, and meditations are extremely useful to decrease circulating cortisol levels and improve “resilience” in stressed subjects (30). All these techniques act by lowering/reducing metabolism, heart rhythm (decreasing of systolic and diastolic blood pressures), respiratory rate (20), and oxygen volume consumption. Indeed, these techniques also allow elimination of carbon dioxide and free radicals and modulation of gene expression, especially with respect to those associated with stress-related health disorders (chronic inflammation, premature aging; decreased brain activity and decreased attention/decision-making functions) (31).

As observed at first session, perceived stress was significantly reduced while stress perception was improved. As previously reported, the PSS-10 test is used for detecting perceived stress, allowing for an understanding of the level at which people judge their lives as unpredictable and overloaded (32). This questionnaire has no cultural or idiographic limits and has a requirement of lower secondary school level to complete it (24). The observation of a decreased total score and in particular the lowering of items 1, 3, and 10 as well as the slight increasing of item 7 would suggest the success of RR training. Moreover, RR training lowered the perception of perceived helplessness (32).

Specifically, the observation of the presence of self-confidence to solve problems has allowed us to conclude that the RR was able to increase perceived self-efficacy.

With respect to BDI-SF, RR training had a strong effect on alleviating depression (*p* = 0.001), but not on single outcomes, as the individual items were not significantly changed. The decision to choose this short version, i.e., BDI-SF, was supported by several previous studies (33, 34). It is important to highlight that RR had a general effect on overall well-being, taking into consideration a non-clinical population herein studied.

The amount of cortisol in saliva samples was considered a useful biomarker for stress conditions, as cortisol represents one of the products of the HPA axis activation (35). Despite the analysis of a small study sample, a trend for a decrease of cortisol in saliva samples was observed upon RR training, which is in line with a previous study (36). The observation that a single RR training decreased saliva cortisol and improved positive effects among individuals (based on data obtained from questionnaires), especially those having high levels of stress, is consistent with the absence of external interfering factors.

NGF and BDNF were previously implicated in stress response at different levels, both in human and animal models (9, 37–39). NGF was found to exert protective actions in nervous, endocrine, immune, and visual systems (40). NGF contribution in psychiatric manifestations has been widely reported by several different authors (41–43). Also, several correlations between BDNF and pathophysiology of brain and psychiatric diseases were found (14, 44, 45).

As a second finding, RR training was found to modulate the levels of NGF and BDNF in saliva and tears. A general increase in NGF levels has been wildly reported as an approaching “unknown” experience, according to the well-known NGF pivotal role in an emotional stress (9, 39). RR training exerted an insignificant decrease of NGF levels in saliva samples upon treatment. At the same time, the amount of saliva BDNF at first session was significantly increased after 2 months of RR training and higher levels of this neurotrophin were detected at the end of RR sessions. These high BDNF levels might be in line with the positive effects played by RR training against stress. An interesting point for future investigation would be to verify whether raising the BDNF with RR could reduce the severity of depression in clinically depressed patients. Recently, stress biomarkers were quantified in saliva, tears, and ocular surface of anxious or psychiatric patients (18, 46, 47). Particularly, cortisol was quantified in tears and its specific receptors were localized on the ocular surface (48–50). Of interest for our study, NGF and BDNF tear expressions were modulated by RR training, and the increase of neurotrophies was statistically significant in the last session. OSDI score was found to be in a normal range between 0 and 25, suggesting that our study subjects were not having dry eye symptoms. Nonetheless, we cannot associate RR training to the benefits brought by NGF or BDNF at the ocular surface level. Taken together, chronic persistence of circulating cortisol levels in stressed subjects may significantly influence cognitive, behavioral, and health body responses (6). In previous studies, altered levels of cortisol, NGF, and BDNF in biological fluids have been associated with stress (5, 9, 10). Our current study highlights the beneficial effects of RR training on mental homeostasis, as assessed by specific questionnaires, and has associated the RR response to changes in BDNF levels in saliva and tears. These promising data sustain the involvement of NGF in stress response, although further studies with larger sample sizes could be carried out.

## Data Availability Statement

The raw data supporting the conclusions of this article will be made available by the authors, without undue reservation.

## Ethics Statement

The studies involving human participants were reviewed and approved by Università Pontificia Salesiana. The patients/participants provided their written informed consent to participate in this study.

## Author Contributions

MZ and FB performed the article drafting and revision, analysis and interpretation of data, and revised the article. RM and BOB performed the data. MC performed the data and interpretation of data. MR performed critical revision of the article for important intellectual content. ED performed the analysis and interpretation of the data and critical revision of the article for important intellectual content. DS and AM performed conception and design, analysis and interpretation of the data, and critical revision of the article for important intellectual content. All authors contributed to the article and approved the submitted version.

## Conflict of Interest

The authors declare that the research was conducted in the absence of any commercial or financial relationships that could be construed as a potential conflict of interest.
